# Impact of dyslipidemia on the severity of symptomatic lumbar spine degeneration: A retrospective clinical study

**DOI:** 10.3389/fnut.2022.1033375

**Published:** 2022-12-13

**Authors:** Zhonglian Huang, Jiechen Chen, Yihong Su, Muxin Guo, Youbin Chen, Yilin Zhu, Guangshuai Nie, Ruitian Ke, Hongjiang Chen, Jun Hu

**Affiliations:** ^1^Department of Orthopaedics, The First Affiliated Hospital, Shantou University Medical College, Shantou, Guangdong, China; ^2^Orthopaedic Medical Research Center, The First Affiliated Hospital, Shantou University Medical College, Shantou, Guangdong, China; ^3^Department of Radiology, The First Affiliated Hospital, Shantou University Medical College, Shantou, Guangdong, China

**Keywords:** intervertebral disc degeneration, endplate inflammation, dyslipidemia, lipoprotein, machine learning

## Abstract

**Background:**

Lumbar intervertebral disc degeneration (IVDD) is an important cause of low back pain or sciatica, and metabolic factors play an important role. However, little is known about the relationship of dyslipidemia to the risk of intervertebral disc degeneration (IVDD). This study aimed to assess the impact of serum lipid levels on the severity of lumbar disc degeneration and to investigate its association with endplate inflammation.

**Methods:**

We conducted a case retrospective study in which a total of 302 hospitalized Chinese patients were recruited, of whom 188 (112 males and 76 females; mean age: 51.66 years) were without underlying disease, while the remaining 114 patients (51 males and 63 females; mean age: 62.75 years) had underlying diseases. We examined fasting serum lipid levels for total cholesterol (TC), triglycerides (TG), low-density lipoprotein cholesterol (LDL-C), and high-density lipoprotein cholesterol (HDL-C). Magnetic resonance imaging (MRI) was used to determine endplate inflammation. Pfirrmann grading and Weishaupt grading were used to evaluate the severity of intervertebral disc degeneration and facet joint degeneration, respectively.

**Results:**

There was no difference in age, gender, and general BMI between the two groups (*P* > 0.05), but there were significantly high levels in TC, LDL-C, and LDL-C/HDL-C (*P* = 0.04, *P* = 0.013, *P* = 0.01, respectively). TG and HDL-C showed no significant difference (*P* = 0.064, *P* = 0.336, respectively). The multivariate logistic regression model showed that age was a risk factor for the occurrence of endplate inflammation. In the group without underlying diseases, age, but not other indicators, was a risk factor for the occurrence of endplate inflammation (*P* < 0.01), In the group with underlying diseases, none of the patient indicators was directly related to the occurrence of endplate inflammation (*P* > 0.05). A nonlinear machine learning model was used to measure the contribution of each factor to the disease outcome and to analyze the effect between the top three contributing factors and the outcome variables. In patients without underlying diseases, the top three factors contributing to the severity grading of intervertebral disc degeneration were age (32.9%), high-density lipoproteins (20.7%), and triglycerides (11.8%). For the severity grading of facet joint degeneration, the top three contributing factors were age (27.7%), high-density lipoproteins (19.4%), and triglycerides (14.6%). For patients with underlying diseases, the top three factors contributing to intervertebral disc degeneration were age (25.4%), BMI (15.3%), and low-density lipoprotein/high-density lipoprotein ratio (13.9%). In terms of degree classification for facet joint degeneration, the top three contributing factors were age (17.5%), BMI (17.2%), and total cholesterol (16.7%).

**Conclusion:**

This study shows that age, high-density lipoprotein, and triglycerides affect the degree of degeneration in patients with symptomatic lumbar degeneration without underlying diseases. Age and BMI are two major factors affecting the severity of degeneration in patients with underlying diseases, and dyslipidemia is a secondary factor. However, there is no clear association between dyslipidemia and the occurrence of endplate inflammation in either group.

## Introduction

Lumbar intervertebral disc degeneration (IVDD) is a common condition that gradually worsens with age. Accompanying intervertebral disc (IVD) herniation, spinal stenosis, and articular facet inflammation, IVDD is the leading cause of low back pain and sciatica. Low back pain, of which approximately 40% of low back pain is caused by IVD degeneration (1–3), is a very common public health problem and a leading cause of workforce loss (4). The cost of treatment is also relatively high, but the related personal and social problems are difficult to quantify (5). A literature review reported that the incidence of chronic low back pain is as high as 36.6% (6). Preventing low back pain in at-risk populations is an important challenge that can help reduce the high healthcare costs associated with treatment and rehabilitation (5). The incidence of sciatica is relatively low, only affecting 2–4% of the population, but is more common in men, between 30 and 50 years of age (7). It is reported that the pain suffered by 87% of patients can be relieved in 3 months (8).

Although IVD has been intensively studied, the exact pathophysiological mechanisms are unknown. The known causes of IVDD are aging, reduction of cells, and changes in ECM composition, which result in the disruption of homeostasis, and changes in terms of function and structure (9). Some important external factors include smoking, trauma, obesity, nutritional and metabolic disorders, abnormal mechanical load, environment, and genetics, which are related to IVDD through the induction of inflammation, apoptosis, secretion of pro-inflammatory cytokines, and autophagy (10–14). Obesity and nutritional and metabolic disorders have been confirmed to be closely related to IVDD. A comprehensive meta-analysis shows obesity to be one of the highest risk factors for IVDD (15). Obesity may trigger IVDD by increasing physical load on the lumbar spine, but metabolic effects are not excludable (16). Most obese patients have abnormally high blood lipid levels (17), and much evidence shows that dyslipidemia can lead to atherosclerosis and related diseases such as coronary heart disease and lower extremity arterial ischemic disease. Some studies suggest that dyslipidemia can lead to lumbar artery atherosclerosis, resulting in decreased blood supply to the lumbar spine (18, 19). When the IVD becomes chronic, the reduction of blood supply can affect the repair of the IVD tissue and accelerate IVDD. Dyslipidemia can also affect IVDD progress through proinflammatory activators. Dysregulated production of cytokine-like hormones (i.e., adipokines), considered key regulators of immune responses and inflammation, has been observed in adipose tissue in obese individuals (20). Obesity-related chronic low-grade inflammation is elevated, with inflammation playing an important role in IVDD pathophysiology. Hypertrophic vertebral marrow adipose tissue has recently been implicated as a source of inflammatory adipokines, which can cause degenerative changes in IVD through metabolic disturbances and the establishment of an initial inflammatory environment (21). The important tissue structure of the nucleus pulposus for nutrient exchange in the intervertebral disc is the cartilaginous endplate. The occurrence of endplate inflammation often leads to a disturbance of nutrient exchange in the IVD and accelerates the degeneration of the IVD (22).

In 2016, the Joint Committee for the Development of Guidelines for the Prevention and Treatment of Dyslipidemia in Adults in China reported that in the past 30 years, the blood lipid level of the Chinese population has gradually increased, and the prevalence of dyslipidemia has increased significantly. A 2012 national survey showed that the prevalence of adult hypercholesterolemia was 4.9%; the prevalence of hypertriglyceridemia was 13.1%, the prevalence of low HDL-C hyperemia was 33.9%, and the overall prevalence of dyslipidemia among Chinese adults was as high as 40.40%, a substantial increase from 2002. Dyslipidemia characterized by elevated low-density lipoprotein cholesterol (LDL-C) or TC is an important risk factor for atherosclerotic cardiovascular disease, as well as other types of dyslipidemia, such as elevated TG. There is also a certain association between the reduction of HDL-C and the increased risk of atherosclerotic cardiovascular disease (23, 24). Elevated serum cholesterol levels in the population will lead to an increase of approximately 9.2 million cardiovascular events in China between 2010 and 2030, indicating that the prevalence of dyslipidemia and related diseases in Chinese adults will continue to increase in the future (25).

To evaluate the relationship between serum lipid levels and the severity of lumbar disc degeneration in symptomatic Chinese patients, we performed a retrospective study of serum lipid levels in symptomatic hospitalized patients. In this study, fasting serum TC, TG, LDL-C, and HDL-C concentrations were measured by biochemical analysis, the presence or absence of endplate inflammation was determined by lumbar MRI, and the Pfirrmann and Weishaupt grading systems were used to evaluate the degree of IVDD and facet joint degeneration. We also investigated whether there was an association between dyslipidemia and endplate inflammation.

## Materials and methods

### Subjects and ethics

This study included inpatients from January 2019 to February 2022 in the Department of Orthopaedics, the First Affiliated Hospital of Shantou University Medical College. Inclusion criteria were: (1) symptoms: low back pain with unilateral or bilateral lower extremity radiculopathy; (2) specific nerve root irritation signs: straight leg raising test and reinforcement test or femoral extension test; (3) neurological deficits: muscle weakness, numbness, or lack of corresponding reflexes (knee twitches or ankle reflexes); and (4) signs of IVDD on magnetic resonance imaging. The exclusion criteria were: (1) patients under the age of 18; (2) cancer; (3) chronic kidney or liver failure; (4) history of spinal trauma and intervertebral space infection; or (5) intervertebral disc surgery. We selected a group of diseases caused by lumbar intervertebral disc degeneration, including lumbar disc herniation, lumbar spinal stenosis, and discogenic low back pain. The diagnosis of lumbar disc herniation, lumbar spinal stenosis, and discogenic low back pain is based on the criteria in the previous literature (26–28). Grouping: Those with diabetes, cardiovascular disease, cerebrovascular disease, immune rheumatism, and abnormal thyroid function were classified as the group with underlying diseases; the remaining patients were classified as the group without underlying diseases. This study was approved by the Ethics Committee of the First Affiliated Hospital of Shantou University Medical College (Reference No.: B-2022-241) and was strictly conducted under international guidelines. Consents were obtained from the participants.

### Data collection

The basic information of patients, including age, gender, height, weight, and disease diagnosis, was routinely collected.

### Blood biochemical tests

Lipid levels of plasma samples from each patient were measured using a fully automated biochemical analyzer following standardized operating procedures (Beckman Coulter AU5800, USA) after an overnight fast of more than 8 hours before surgery, as well as patients who underwent surgery. Other blood tests were also performed on all patients, including liver and kidney function, blood glucose level, etc.

### Radiological assessments

**FIGURE 1:**
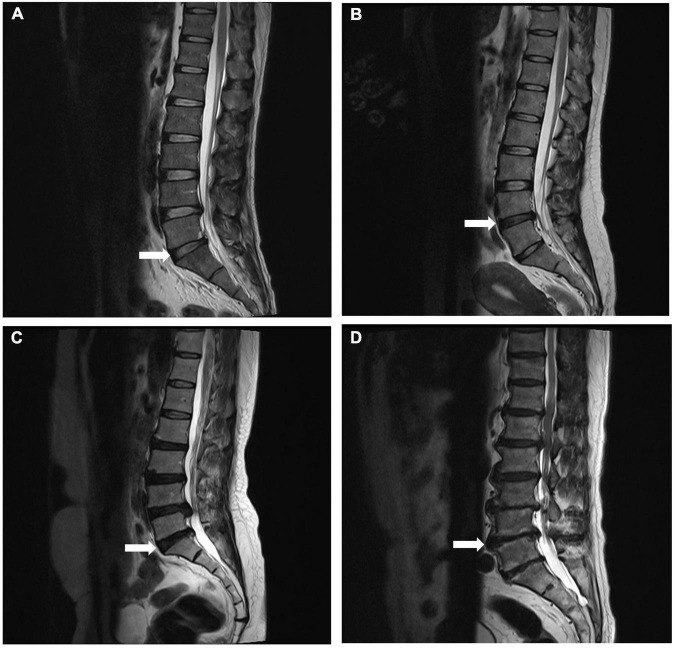
Representative MRI images used for Pfirrmann grading system of lumbar disc degeneration. Midsagittal T2-weighted views showing grade 2 **(A)**, grade 3 **(B)**, grade 4 **(C)**, and grade 5 **(D)** degeneration of the lumbar disc.

**FIGURE 2:**
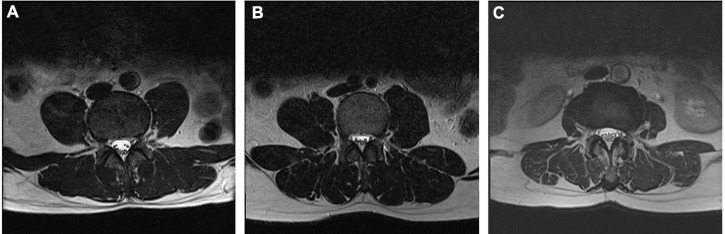
Representative MRI images used for Weishaupt grading system of lumbar facet joint degeneration. Axial T2-weighted views showing grade 1 **(A)**, grade 2 **(B)**, and grade 3 **(C)** osteoarthritis of the lumbar facet joint.

The lumbar spine was scanned with a 1.5 Tesla MRI scanner (Siemens, Avanto, Germany) following standardized protocols. On T2-weighted midsagittal images, the degree of degeneration in the entire lumbar segment (L1/2 to L5/S1) was assessed by the Pfirrmann grading (29) (Figure 1); on T2-weighted cross-sectional images of the IVD, the Weishaupt grading was used to evaluate the degree of degeneration of the facet joints in the lumbar segment (L2/3 to L5/S1) (30) (Figure 2). The segment with the most severe degeneration was used for evaluation. All images were independently measured and evaluated by a radiologist (Ruitian Ke) and orthopedic surgeon (Zhonglian Huang) with over 10 years of experience. If there was a discrepancy in the radiological assessment between the radiologist and the surgeon, the radiologist made the final decision after consulting the surgeon. The presence or absence of endplate inflammation was directly determined based on the imaging results.

The intra-class correlation coefficient (ICC) was used to determine the inter-observer reliability of radiometric measurements, with higher values indicating better reliability. ICC values of 0.40–0.59 were considered fair, 0.60–0.74 were considered good, and ICC values greater than 0.75 were considered excellent (31). In our study, ICC for Pfirrmann grading was 0.809, and the ICC for Weishaupt grading was 0.711 (see Supplementary Table 1).

### Statistical analyses

SPSS 28.0 (SPSS Inc., Chicago, Illinois, USA) was used for the statistical description of the influencing factors between and within the two groups (no underlying disease group vs. underlying disease group). Specifically, serum lipids were compared between groups using the independent samples *t*-test. Quantitative data were expressed as means ± SD. The Kendall correlation was used to analyze the relationship between between-group/within-group factors and disease outcomes. R 1.4 software was used to construct a multivariate logistic regression model to study the association between the disease outcome (binary variable) of endplate inflammation and potential influencing factors. The OR value (odds ratio) and its confidence interval (95% CI) were used to evaluate the potential impact risk factors for endplate disease outcomes. All the above hypothesis tests were two-sided, and the test level have an α = 0.05. We used JMP Pro 16 software to perform two independent sample mean power tests between influencing factors and diseases on the number of cases in this study. Specifically, 302 patients were included, and the test type was set as bilateral, the first type of error probability α = 0.05, power value calculated: 1-β both exceed 0.9, suggesting that, the sample size in this study is sufficient.

### Interpretive machine learning analytics

A nonlinear machine learning model (eXtreme Gradient Boosting: XGBoost) was used to fit the influencing factors and disease outcome variables (facet joint degeneration, IVDD) into one of two groups (no underlying disease group vs. underlying disease group). R^2^ and RMSE (root mean square error) were used as the evaluation criteria for the degree of fit. SHAP (SHapley Additive exPlanations) was used to measure the contribution of each factor to the disease outcome and analyze the effect between the top three contributing factors on the outcome variable and the outcome variable (the SHAP value of the important factor), SHAP > 0 means promoting effects on disease outcomes and vice versa. A Fourier function was used to fit SHAP values and draw nonlinear effect curves between important influencing factors and disease outcomes.

## Results

### General characteristics

The no underlying disease group included 188 subjects (112 men and 76 women) with a mean age of 51.66 years and mean body mass index (BMI) of 23.54 kg/m^2^. The underlying disease group consisted of 114 subjects (51 males and 63 females) with a mean age of 62.75 years and a mean BMI of 24.06 kg/m^2^. As summarized in Table 1, there were no differences in age, sex, and body mass index between the two groups. However, in general, the patients in the underlying disease group were slightly older and had a slightly higher BMI index.

**TABLE 1:** Baseline characteristics of participants (*n* = 302).

Variables	Group 1 (no underlying diseases) (*n* = 188)	Group 2 (underlying diseases) (*n* = 114)	*P*-value
Gender (M/F)	112/76	51/63	0.516
Age (years)	51.66 ± 15.69	62.75 ± 11.12	0.231
Range (years)	19–87	19–90	
BMI (kg/m^2^)	23.54 ± 3.0	24.06 ± 3.2	0.862

Data on age and BMI are shown as the means ± SD. BMI, body mass index.

### Comparison of blood lipid levels and other biochemical indicators between the two groups of patients

As summarized in Table 2, TC, LDL-C, and LDL-C/HDL-C ratio were significantly different between the two groups (*P*-values were 0.04, 0.013, 0.01, respectively). The levels of TC and LDL-C in the group with underlying diseases were higher, and the LDL-C/HDL-C ratio was higher, indicating that the imbalance of blood lipid components was more dramatic, and the proportion of LDL-C was greater. Although HDL-C levels did not differ significantly between the two groups, patients with underlying disease tended to be lower. There is no statistical difference in the levels of phosphocreatine isoenzyme, phosphocreatine kinase, glutamic oxaloacetic transaminase, glutamic pyruvic transaminase, glutamyl transpeptidase, monoamine oxidase, total bilirubin, direct bilirubin, indirect bilirubin, glucose, urea nitrogen, and uric acid on intervertebral disc degeneration, facet joint degeneration, and endplate inflammation (*P* > 0.05) (see Supplementary Tables 2, 3).

**TABLE 2:** Concentrations of lipids (mmol/L) and LDL-C/HDL-C ratio in the two groups.

Serum lipids	No underlying diseases (*n* = 188)	Underlying diseases (*n* = 114)	*t*- value	*P*- value
TC (mmol/L)	5.4318 ± 1.09	5.7144 ± 1.26	–2.062	0.040
TG (mmol/L)	1.4921 ± 0.88	1.6911 ± 0.94	–1.857	0.064
LDL-C (mmol/L)	3.3908 ± 0.77	3.6298 ± 0.88	–2.486	0.013
HDL-C (mmol/L)	1.3714 ± 0.94	1.2837 ± 0.33	0.964	0.336
LDL-C/HDL-C	2.6996 ± 0.79	2.9473 ± 0.85	–2.579	0.010

Data are shown as the means ± SD and analyzed using independent-sample *t*-tests.

### Contribution of individual influencing factors to the degree of degeneration

**FIGURE 3:**
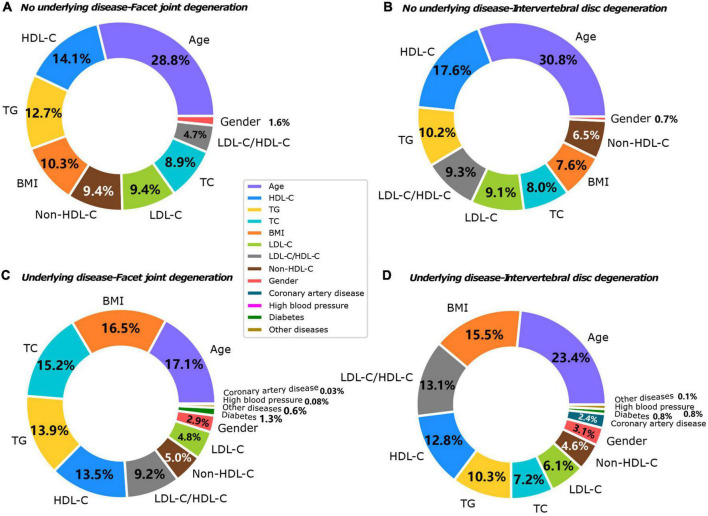
Contributing factors to IVDD in both groups. **(A)** No underlying disease-facet joint degeneration. **(B)** No underlying disease-IVDD. **(C)** Underlying disease-facet joint degeneration. **(D)** Underlying disease-IVDD.

**FIGURE 4:**
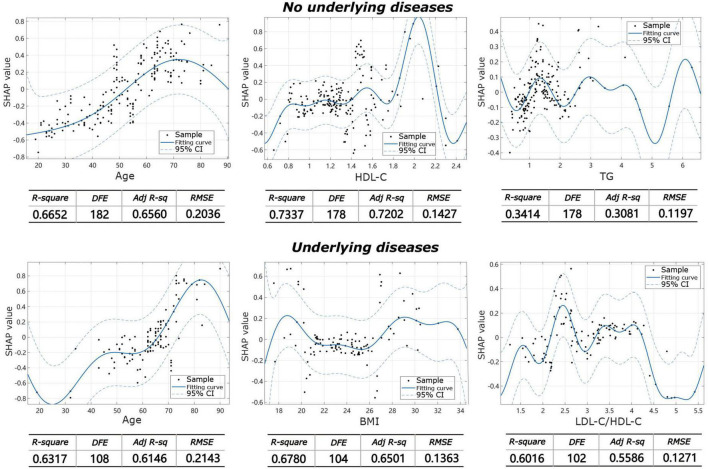
Fitting effect curves show the association between SHAP values and contributing factors in IVDD.

**FIGURE 5:**
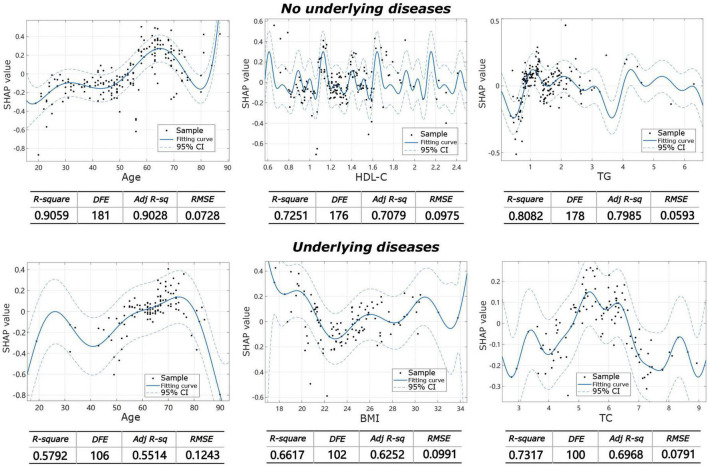
Fitting effect curves showing the association between SHAP values and contributing factors in facet joint degeneration.

The machine learning model showed that the influencing factors of the group without underlying diseases included age, gender, BMI, TC, TG, HDL-C, LDL-C, and LDL-C/HDL-C. Influencing factors in the group with underlying diseases included age, gender, BMI, TC, TG, HDL-C, LDL-C, and LDL-C/HDL-C, diabetes, coronary heart disease, hypertension, and other systemic diseases. The model analysis showed that in patients without underlying diseases, the top three contributing factors for the severity of IVDD were age (32.9%), HDL-C (20.7%), and TG (11.8%). For facet joint degeneration severity, the top three contributing factors were age (27.7%), HDL-C (19.4%), and TG (14.6%). In patients with underlying disease, the top three contributing factors for IVDD severity were age (25.4%), BMI (15.3%), and LDL-C/HDL-C (13.9%), and for facet joint degeneration severity, the top three contributing factors were age (17.5%), BMI (17.2%), and TC (16.7%) (Figure 3). The fitting effect curves showed that among patients age was a risk factor for disease outcome, and there was a gradual increase in disease severity with age (Figures 4, 5). However, HDL-C and TG failed to show such a trend (Figures 4, 5).

### Relationship between various factors and endplate inflammation

**FIGURE 6:**
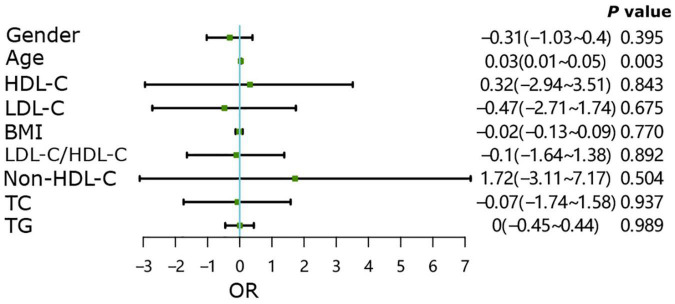
Contributing factors and endplate inflammation in patients without underlying diseases.

**FIGURE 7:**
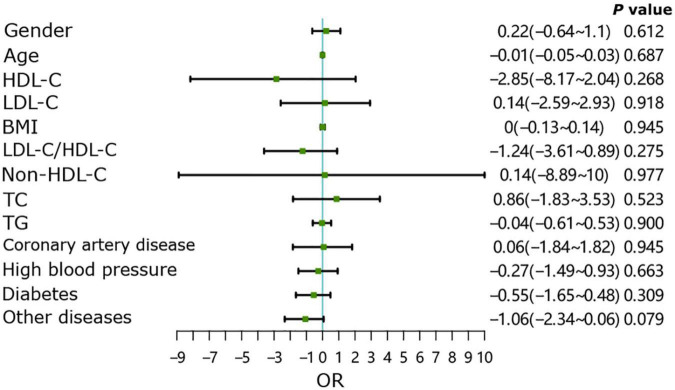
Contributing factors and endplate inflammation in patients with underlying diseases.

As shown in Figure 6, in the group without underlying diseases, age may be a risk factor for the occurrence of endplate inflammation (*P* < 0.01), with other indicators showing no effect on the occurrence of endplate inflammation (*P* > 0.05). In the group with underlying diseases, all patient indicators were not directly related to the occurrence of end plate inflammation (Figure 7, all *P* > 0.05).

## Discussion

In addition to its well-known impact on the cardiovascular system, the impact of dyslipidemia on other systems has also attracted increasing attention. An epidemiological survey in Finland found a correlation between TC, LDL-C, TG levels, and sciatica in men (32), and a follow-up study reported that elevated TC and TG were predictive of radiation-induced low back pain in the working population (33). In a large prospective cohort study in Norway that included women 30–69 years of age, TC levels showed no correlation among women without low back pain at baseline, although a weaker relationship was observed between women with low back pain at baseline and men without low back pain at baseline. In men with low back pain at baseline, there is an inverse association with HDL-C (34), and a large cross-sectional survey of adults in Japan between the ages of 40 and 64 found that low HDL-C and high LDL-C/HDL-C ratios are associated with low back pain (35). These all indicate that there is a clear correlation between dyslipidemia and lumbar degenerative diseases. However, the roles of blood lipid components in the results of these studies are not consistent, which may be due to differences in the living habits of different races and regions. At the same time, the ratio of blood lipid components, such as the ratio of LDL-C/HDL-C, is also considered to be an important indicator that requires our attention. An Italian case-control study found that patients with symptomatic lumbar disc herniation exhibited statistically significant higher TG concentration (*P* = 0.02) as well as TC concentration (*P* = 0.01), possibly due to pathological factors involving lumbar disc degeneration (36). A case-control study in China found that the ratios of TC/HDL-C and LDL-C/HDL-C are associated with disc herniation and that patients with higher serum LDL-C levels have a higher risk of developing disc herniation, suggesting that serum lipid levels may be a useful predictor of intervertebral disc degeneration in the Chinese population (37). However, none of the above studies evaluated the effect of lipid components and their proportions on the severity of degeneration. The only study analyzing the severity of disc degeneration found that elevated TG and abdominal obesity had a more pronounced effect on the degree of disc degeneration and appeared to play a crucial role in the development of lumbar IVDD. However, the study involved a small group of patients and was limited to the phenotypes of “healthy lipids but obesity” and “dyslipidemia but not obesity” (38).

In the present study, we analyzed the influence of various factors on the severity of IVD and facet joint degeneration through a machine learning model, and found that in the group without underlying diseases, in addition to age, which is recognized as an important factor, HDL-C and TG are the second most important factors. Some of the above findings are in partial agreement with ours. Regarding the results of blood lipid levels in patients with and without underlying disease, we further analyzed the Kendall correlation and found that triglyceride levels were significantly different in the two groups concerning the severity of intervertebral disc and facet joint degeneration (Kendall, 0.995, 95%CI, 0.994–0.996). Combined with the results of the above contribution, our study further indicates that TG may have a more important role in the severity of IVD or facet joint degeneration. The exact pathophysiological mechanism underlying the link between serum lipid levels and IVDD is unknown. It has been hypothesized that dyslipidemia may be associated with lumbar vertebral atherosclerosis. TG levels are known risk factors for atherosclerosis (39). Atherosclerotic plaques and calcifications tend to develop in or around the lumbar artery branches, resulting in segmental lumbar artery stenosis or occlusion (40, 41). This hinders the nutrient supply of the corresponding lumbar segment and leads to IVDD. Atherosclerosis can therefore block the blood supply to the corresponding lumbar region, resulting in IVDD and surrounding tissue damage (42). In addition, there may be other potential pathways for TG to cause or promote disc degeneration. IVDD is thought to be partially mediated by infiltrating inflammatory cells (10), and the presence of pro-inflammatory cytokines further exacerbates disc degeneration. Hypertriglyceridemia may mediate IVD apoptosis and matrix catabolism primarily through the ERK pathway (16). Our findings suggest that HDL is a protective factor against IVDD, which is consistent with its protective role in the cardiovascular system. Raising HDL concentrations may help reduce IVDD.

For the underlying disease group, we included the existing underlying diseases as influencing factors into the model for analysis and showed that age and BMI had the greatest impact on the degree of IVDD and facet joint degeneration, which is consistent with other studies (43–46). Of note, we found that the proportion of each underlying disease is minor. Our analysis may be attributed to the fact that other factors are continuous variables, and the inclusion of basic diseases is a dichotomous variable with or without, resulting in a small contribution to the results. This is also the limitation of this study. Subsequent studies are expected to quantify the underlying disease and are expected to obtain a more accurate assessment.

Endplate inflammation (Modic change) is a signal change in the vertebral endplates and adjacent bone marrow is visible on MRI, reflecting microscopic changes in endplate tissue biochemistry, an early manifestation of endplate degeneration. Endplate inflammation is common in the clinic, but the exact mechanism remains unknown. The relationship between it and low back pain has been extensively studied and most investigators believe there is a connection (47–49). The occurrence of endplate inflammation is related to multiple factors including genetics, smoking, being overweight and physical labor. Three types of endplate inflammation have been described based on their appearance in T1-weighted and T2-weighted images. Modic type 2 changes are associated with the decreased height of IVD and increased fat mass index, suggesting that Modic changes may be related to metabolic components (50). Two recent studies have directly investigated the relationship between blood lipids and endplate inflammation. One study found that a significant correlation was observed between Modic change and TG, which may be one of the factors that accelerate cervical degeneration (51). Another study examined biomarkers of Modic change using a metabolomic approach and found that they were associated with the mean diameters of very low-density lipoprotein (VLDL)/low-density lipoprotein (LDL) and cholesteryl ester/phospholipid in large LDL particles. Statistically, a reduction in the mean diameter of VLDL may lead to Modic change (52). The results of our study show that in the group without underlying diseases, age may be a risk factor for the occurrence of endplate inflammation (*P* < 0.01), with other indicators, examined being unrelated to the occurrence of endplate inflammation (*P* > 0.05). In the future, we need to either increase the sample size or use related assays, such as metabolomics, to further explore the effects of blood lipid components and their contributions to endplate inflammation. Many studies have investigated the relationship between different types of endplate inflammation and low back pain, but few reports on quantitative evaluation of endplate inflammation. Amir Jamaludin developed an automatic detection system for quantitative assessment of the severity of endplate inflammation, which is similar to the assessment results of radiologists (53). However, it has not been widely used due to little research on quantitative evaluation criteria for the severity of endplate inflammation. For intervertebral disc degeneration, the Pfirmann scoring system is widely used for the quantitative assessment. There have been many studies on the degree of intervertebral disc degeneration through machine learning, which can achieve a very accurate assessment of the degree of degeneration (54, 55).

In conclusion, our study used a new method (i.e., machine learning) to assess the impact of lipid components on the severity of intervertebral disc degeneration. Limitations of this study include that the design of the study was cross-sectional and could only demonstrate the effect, rather than causation, between specific risk factors on the severity of IVDD. While some retrospective information can be gleaned from medical records, retrospective studies leave causality undetermined and cannot rule out other factors that may have influenced the course of IVDD. Subsequent large-scale longitudinal follow-up observational and intervention studies are needed to obtain more convincing results. Thyroid function and HbA1C are possible related factors affecting lipid metabolism, however, based on the local medical insurance policy in China, thyroid function and HbA1C test are not routine tests for inpatients. Only patients diagnosed with thyroid dysfunction or diabetes are requested to perform thyroid function and HbA1C test. In our study, there were only two patients with hyperthyroidism. Of note, considering that all recruited cases were from non-iodine deficient regions, the incidence rate of thyroid subclinical abnormalities is low, we believe that thyroid function is not the main factor in the present study. Given that the causal relationship between HbA1C and lipids is still uncertain (56–58) especially in non-diabetic patients, we did not analyze HbA1c as an influencing factor of dyslipidemia. Additionally, in the underlying disease group, we divide the existing complications into four types: hypertension, diabetes, coronary heart disease, and other diseases. For some complications with a small number of cases except for the top three, the statistical results of all cases combined cannot clarify the impact of these specific complications. Although the severity of each complication will have different effects on the results, we assign the impact values of these complications as 1 (with = 1) and 0 (without = 0) in the statistical data because this is a cross-sectional study to analyze whether these complications have an impact on the outcome. Some researchers have developed the application of machine learning to quantitative assessment of intervertebral disc and facet joint degeneration, which may eliminate subjective errors, but its analysis application is still limited to the evaluations of the researcher. Therefore, we prefer to use the traditional evaluation method, and the statistical results of ICC show that our observation error is acceptable. Although the power calculations are higher than 90% (see Supplementary Table 4), the overall sample size still is small, the risk of bias still exists. In future studies, we will expand the sample size to better explain the exact relationship between serum lipids and the severity of IVDD.

## Conclusion

Our findings suggest that HDL-C and TG are closely related to the severity of disc degeneration and that increasing the level of HDL or lowering the level of TG can help reduce the severity of IVDD. The present study does not support a correlation between dyslipidemia and the occurrence of terminal plate inflammation, therefore further research is needed.

## Data availability statement

The original contributions presented in this study are included in the article/Supplementary material, further inquiries can be directed to the corresponding author.

## Ethics statement

The studies involving human participants were reviewed and approved by Ethics Committee of The First Affiliated Hospital of Shantou University Medical College. The patients/participants provided their written informed consent to participate in this study.

## Author contributions

JH and ZH conceived the study, participated in the design, and drafted the manuscript. JC, YS, MG, and YC collected the data. YZ, RK, GN, and HC performed the statistical analysis, and prepared the tables and figures. All authors have read and approved the manuscript submission in its current version.
